# A prospective pilot study of urine microRNA as a biomarker of urinary radiation toxicity following MRI-guided prostate stereotactic body radiotherapy

**DOI:** 10.3389/fonc.2026.1951947

**Published:** 2026-10-07

**Authors:** Urszula Smyczyńska, Zuzanna Nowicka, Patrick Doyle, Heejoo Kang, Maeve Dillon-Martin, Anthony D’Amico, Dipanjan Chowdhury, Wojciech Fendler, David Kozono, Jonathan E. Leeman

**Affiliations:** 1 Department of Biostatistics and Translational Medicine, Medical University of Lodz, Lodz, Poland; 2 Department of Radiation Oncology, Dana-Farber Cancer Institute/Brigham and Women’s Hospital, Boston, MA, United States

**Keywords:** biomarkers, microRNAs, prostate cancer, stereotactic body radiotherapy, urinary toxicity

## Abstract

**Introduction:**

Urinary toxicities are common following prostate radiotherapy and can impair quality of life. New biomarkers are needed that can predict the risk of urinary toxicity following MRI guided stereotactic body radiotherapy (MR-SBRT) to assist with selection of the most appropriate radiotherapy treatment approach. We aimed to identify urine miRNA biomarkers of radiation-induced urinary toxicities.

**Methods:**

Ten patients undergoing MR-SBRT were enrolled on a prospective biorepository protocol and urine specimens were collected before, at the final fraction and 2-4 months following radiotherapy. RNA was isolated and microRNA sequencing was performed followed by qPCR for confirmation of microRNA expression changes. Differences in microRNA expression were assessed with regard to timepoint, cancer risk category and urinary toxicity.

**Results:**

Both sequencing and qPCR showed that expressions of miR-26a-5p and miR-95-3p decrease during MR-SBRT and increase during early follow up. miR-204-3p and miR-192-5p changed differently in patients with and without urinary radiation toxicity. Expression of miR-375-3p was lower in patients with higher risk category (NCCN unfavorable intermediate to high risk or recurrent/metastatic) at all timepoints and decreased during and after MR-SBRT, especially in patients with a lower risk category (NCCN low to favorable intermediate risk) of prostate cancer.

**Discussion:**

Urine microRNA analysis is feasible and may identify biomarkers linked to radiation-induced urinary toxicity and prostate cancer severity. The findings of this pilot study are limited by the sample size and should be prospectively validated.

## Introduction

Acute and chronic urinary side effects are common following prostate radiotherapy. Stereotactic body radiotherapy (SBRT) in particular may result in higher risk of some urinary toxicities (1). Although clinical factors, including bladder volume, radiotherapy technique and dose-volume metrics may aid in identifying patients at risk, they do not account for biological determinants of responses to radiotherapy. There is a need for biomarkers to predict which patients will develop urinary toxicity, to select the most appropriate radiotherapy approach and potentially provide early intervention. Stereotactic MRI guided adaptive radiotherapy (MR-SBRT) is a new treatment approach that shows promise in reducing the urinary toxicities of radiation (2, 3).

MicroRNAs are short, non-coding regulatory RNA molecules that orchestrate stress responses. They are known to be released into body fluids, including urine, under physiological as well as pathologic conditions such as resulting from radiation injury (4, 5). They are also dysregulated in malignancy and urine microRNAs may aid in non-invasive diagnosis of urologic cancers (6). A score calculated based on germline single nucleotide polymorphisms that disrupt miRNA binding to regulatory regions was recently suggested to predict RT-associated genitourinary toxicity (7).

We hypothesized that bladder urethral injury following MR-SBRT results in the release of specific miRNAs into the urine which can be measured and utilized as an early biomarker of urinary radiation toxicity.

## Materials and methods

### Patients

Patients were enrolled on the BWH Radiation Oncology All-Department Biorepository to Accelerate New Discoveries (BROADBAND), an institutional database and biorepository for patients undergoing radiotherapy. Sample and data collection were approved by the Mass General Brigham Institutional Review Board on 10/7/2021 under protocol 2016P001582 and secondary use of samples and data were approved under protocol 2021P002198.

Eligible participants included patients with a diagnosis of prostate cancer who were planned to receive MR-SBRT.

All study samples were collected under locally approved institutional review board protocols after obtaining informed consent from study participants. All procedures were conducted according to the principles expressed in the Declaration of Helsinki and in accordance with institutional guidelines and regulations.

The primary endpoint was longitudinal change in urine miRNA expression during MR-SBRT and early follow-up and its association with urinary toxicity. Secondary endpoints were the relationship between miRNA expression and prostate cancer risk category and evaluation of radiation-related urinary toxicity.

### Radiation therapy

Patients were treated with prostate directed radiotherapy to a dose of 36.25 Gy in 5 fractions performed every other day. MR-SBRT was performed as previously described (8) with daily online adaptive planning. Target volumes included the prostate and seminal vesicles. The PTV margin was 3mm circumferentially. The urethra and bladder neck were contoured in all cases with the assistance of an MRI. Patients were prepared with a half full bladder by drinking 6oz of water 30 min prior to treatment. Details of this RT protocol were previously described by Leeman et al. in (8).

### Samples and RNA isolation

Urine samples were collected from 10 patients with prostate cancer at three timepoints. The first collection took place at the time of simulation for the purpose of planning MR-SBRT. Then, patients had the treatment administered in 5 fractions, and the second sample was taken at the 5^th^ fraction visit before radiation dose delivery, thus at cumulative dose of 29 Gy (this sample is later referred to as mid-treatment one). The third urine sample was collected 2–4 months after radiotherapy ended when clinicians evaluated if radiation toxicity effects occurred (**Figure 1**). Urine samples were collected in clinic in BD Vacutainer urine collection cups (Becton Dickinson) and aliquoted into 15 mL conical vials (Corning) using serological pipettes. Samples were then centrifuged at 3000 x g for 15 minutes at room temperature and 10 mL supernatant was transferred to a new conical vial for storage at -80 °C. RNA was isolated from 6 ml of urine using the exoRNeasy Maxi (QIAGEN) according to manufacturer’s instructions.

**Figure 1:**
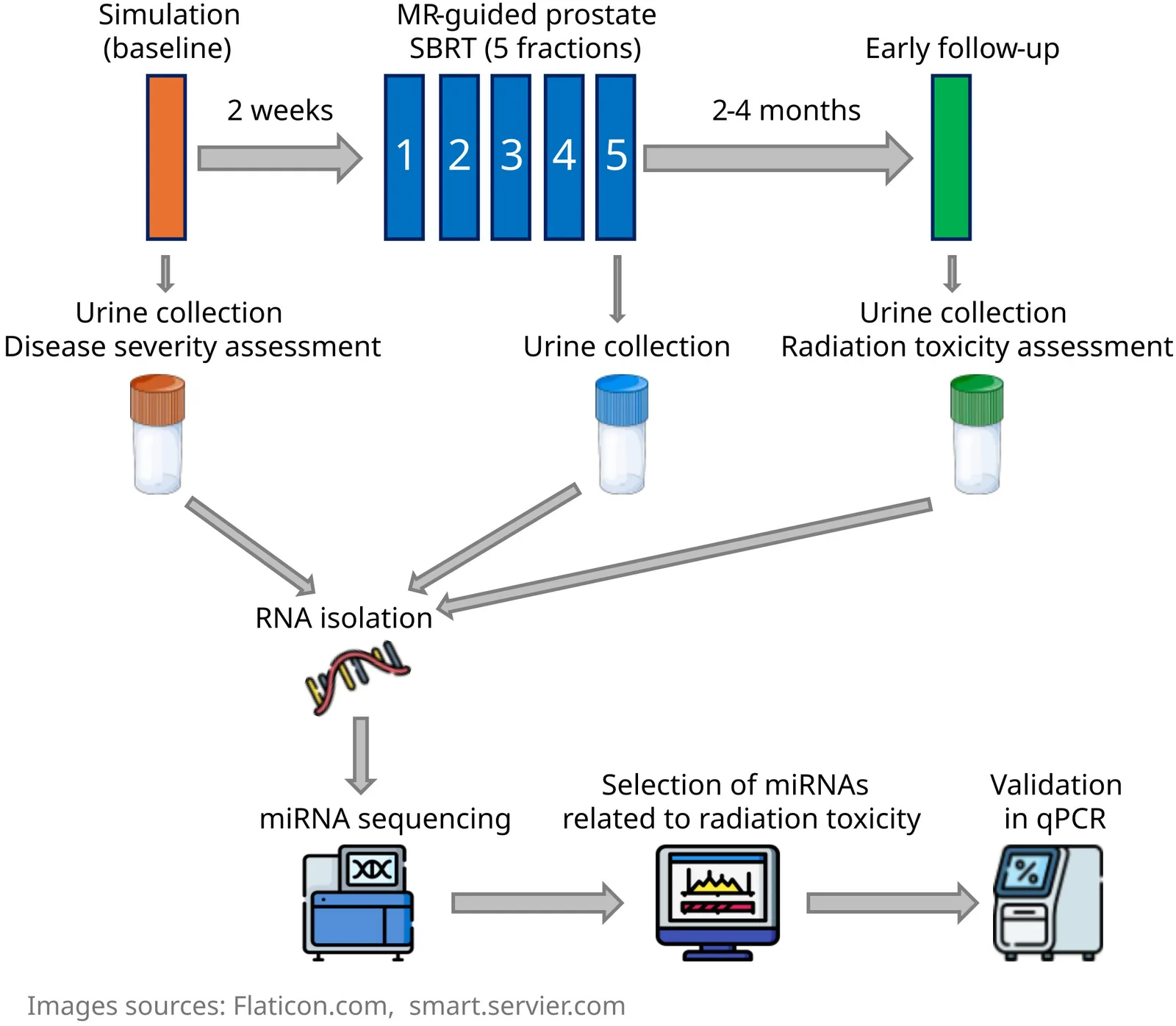
Scheme of the experimental design.

### miRNA sequencing

miRNA sequencing libraries were prepared from 10 ng of input RNA using the QIAseq miRNA Library Kit (QIAGEN). In addition to standard adapters, unique molecular identifiers (UMI) were added at the reverse transcription step before 19 cycles of amplification by PCR and sequencing on a NextSeq (Illumina Inc.) sequencing instrument. Details of bioinformatic miRNA sequencing analysis are described in Supplementary Methods.

After miRNA quantification, based on the DE results, we prioritized miRNAs for confirmatory assays with qPCR according to the following criteria:

- Group 0: reference miRNAs identified by NormiRazor (9). As candidate normalizers, we considered every miRNA with at least 100 CPM in every sample.
- Group 1: miRNAs that at baseline differed between patients with and without urinary radiation toxicity.
- Group 2: miRNAs that differed between patients with and without urinary radiation toxicity both mid-treatment and post-treatment.
- Group 3a: miRNAs that differed in at least 2 between timepoints comparisons in patients with urinary radiation toxicity.
- Group 3b: miRNAs that differed in at least 2 between timepoints comparisons in patients without urinary radiation toxicity.
- Group 4: selected miRNAs that differed between patients with and without radiation toxicity mid- or post-treatment

### qPCR

miRNA expression was measured in duplicate using real-time PCR as described previously (10). In short, each sample with sufficient material available was reverse transcribed into cDNA and its expression was measured using the miRCURY LNA miRNA PCR (QIAGEN). To evaluate the reverse transcription reaction, a spike-in (UniSp6) was used. Normalization was performed using reference miRNA selected in the miRNA sequencing experiment (let-7g-5p, **Supplementary Figure 6** in **Data Sheet 1**) that was detected in all samples with qPCR. miRNAs with missing values (Ct>38) in at least half of samples were excluded from downstream analysis.

For each miRNA, relative expression was normalized to let-7g-5p (ΔCt = Ct_let-7g − Ct_miRNA, such that higher values indicate higher expression). A linear mixed model was fitted per miRNA using the MixedLM function of the Python statsmodels library, with fixed effects of timepoint, radiation toxicity, their interaction, and disease severity, and a random intercept for patient to account for repeated sampling: ΔCt ~ timepoint * radiation_toxicity + severity. Reference categories were baseline (timepoint), no toxicity (radiation toxicity), and grade 0 (severity). For each fixed effect we report the coefficient (β, ΔCt scale) with its 95% confidence interval; p-values were adjusted across miRNAs within each effect using the Benjamini-Hochberg false-discovery-rate procedure.

### Pathway analysis

MiRNA Enrichment and Annotation Analysis (miEAA) was performed using web-based application (11) – an over-representation analysis tool and was done using KEGG pathways and Benjamini-Hochberg correction. Two-sided p value < 0.05 was used to denote significance.

### Toxicity and disease severity assessment

Treatment related toxicity assessments were performed by a physician in accordance with CTCAE v5. For the purposes of this analysis, radiation toxicity was defined as any grade 2 or higher toxicity during or within 3 months of completion of radiotherapy. Prostate cancer disease severity was defined according to NCCN risk stratification criteria (12). Low disease severity was defined as NCCN low to favorable intermediate risk and high disease severity was defined as NCCN unfavorable intermediate to high risk or recurrent/metastatic cancer.

## Results

From 11/2021-5/2022, ten patients with prostate cancer were enrolled. Baseline patient characteristics are shown in **Table 1** and **Supplementary Table 1** in **Data Sheet 9**. Five (50%) were treated for low severity prostate cancer and five (50%) were treated for high severity prostate cancer as defined according to NCCN risk categorization. Four patients (40%) experienced urinary radiation toxicity which in all cases was CTCAE grade 2 urinary frequency or urgency requiring prescription medication; these patients received tamsulosin in response to urinary symptoms.

**Table 1:** Baseline patient characteristics.

Variable	Group/statistic	All patients	Toxicity group	Toxicity-free group
Age	Median (range)	71 (56–89)	70 (56–89)	71 (59-79)
Gleason grade	1	1 (10%)	0	1
	2	4 (40%)	2	2
	3	3 (30%)	1	2
	4	2 (20%)	1	1
NCCN risk group	Low	1 (10%)	0	1
	Favorable intermediate	4 (40%)	2	2
	Unfavorable intermediate	1 (10%)	0	1
	High	3 (30%)	1	2
	Recurrent/Metastatic	1 (10%)	1	0
Received androgen deprivation therapy	Yes	5 (50%)	3	2
	No	5 (50%)	1	4
Prostate volume (cc)	Median (range)	32.8 (19.9-89.1)	32.8 (21.1-67.4)	47.7 (19.9-89.1)
Bladder V36.25 (cc)	Median (range)	2.0 (0-4.8)	1.6 (0.4-3.4)	2.6 (0-4.8)
Bladder V32.62 (cc)	Median (range)	9.6 (0.2-15.3)	9.1 (3.0-11.2)	9.9 (0.2-15.3)
Bladder V38.06 (cc)	Median (range)	0 (0-0.1)	0.05 (0.0-0.1)	0.0 (0.0-0.06)
Urethra V38.78 (cc)	Median (range)	0 (00)	0 (0-0)	0 (0-0)
Urinary medications used during treatment	Tamsulosin	4 (40%)	4	0
	None	6 (60%)	0	6
Baseline AUA/IPSS score	Median (range)	9 (1-12)	9 (4-12)	8 (1-11)
	Not available (N)	1	1	0

In total, 27 samples contained enough material to perform miRNA sequencing (**Data Sheet 1**), including 10 samples from the baseline, 10 collected mid-treatment, and 7 collected post treatment (two patients have not attended follow-up – one in toxicity and one in toxicity-free group, and one in toxicity-free group did not have sample sequenced due to low quality of material). Total number of reads ranged from 4614 to 155,706, averaging 30,143 in miRge3.0 mapping with UMI deduplication (counts in **Data Sheet 2**). When nf-core/smrnaseq without UMI correction was used, the mean number of counts increased to 45,639. This moderate increase confirms that obtained low number of reads were not a result of mapping error. In further analysis, we used mapping by miRge3.0. Filtration resulted in inclusion of 111 miRNAs in differential expression analysis (**Data Sheet 3**).

Differences in miRNA abundance between urinary radiation toxicity and toxicity-free patients were the most pronounced mid-treatment with 11 differentially expressed miRNAs. At baseline and post-treatment, these groups differed nominally in expression of 3 and 7 miRNAs, respectively (**Figures 2A**; **Supplementary Figure 1** in **Data Sheet 1, Data Sheet 4**). The group with urinary radiation toxicity experienced changes in 8 miRNAs from the baseline to mid-treatment, 14 miRNAs from mid-treatment to post-treatment and 9 miRNA from baseline to post-treatment (**Figures 2B**; **Supplementary Figure 2** in **Data Sheet 1, Data Sheet 5**). In the toxicity-free group, expression of 7 miRNAs changed from the baseline to mid-treatment, of the 7 miRNAs from mid-treatment to post treatment and 5 miRNA from baseline to post-treatment. (**Figures 2C**; **Supplementary Figure 3** in **Data Sheet 1**, **Data Sheet 6**). Expression of these miRNAs did not correlate significantly with AUA/IPSS at baseline (**Supplementary Table 2** in **Data Sheet 1**) in 9 patients whose scores were reported. This analysis resulted in selection of following miRNAs for qPCR validation:

**Figure 2:**
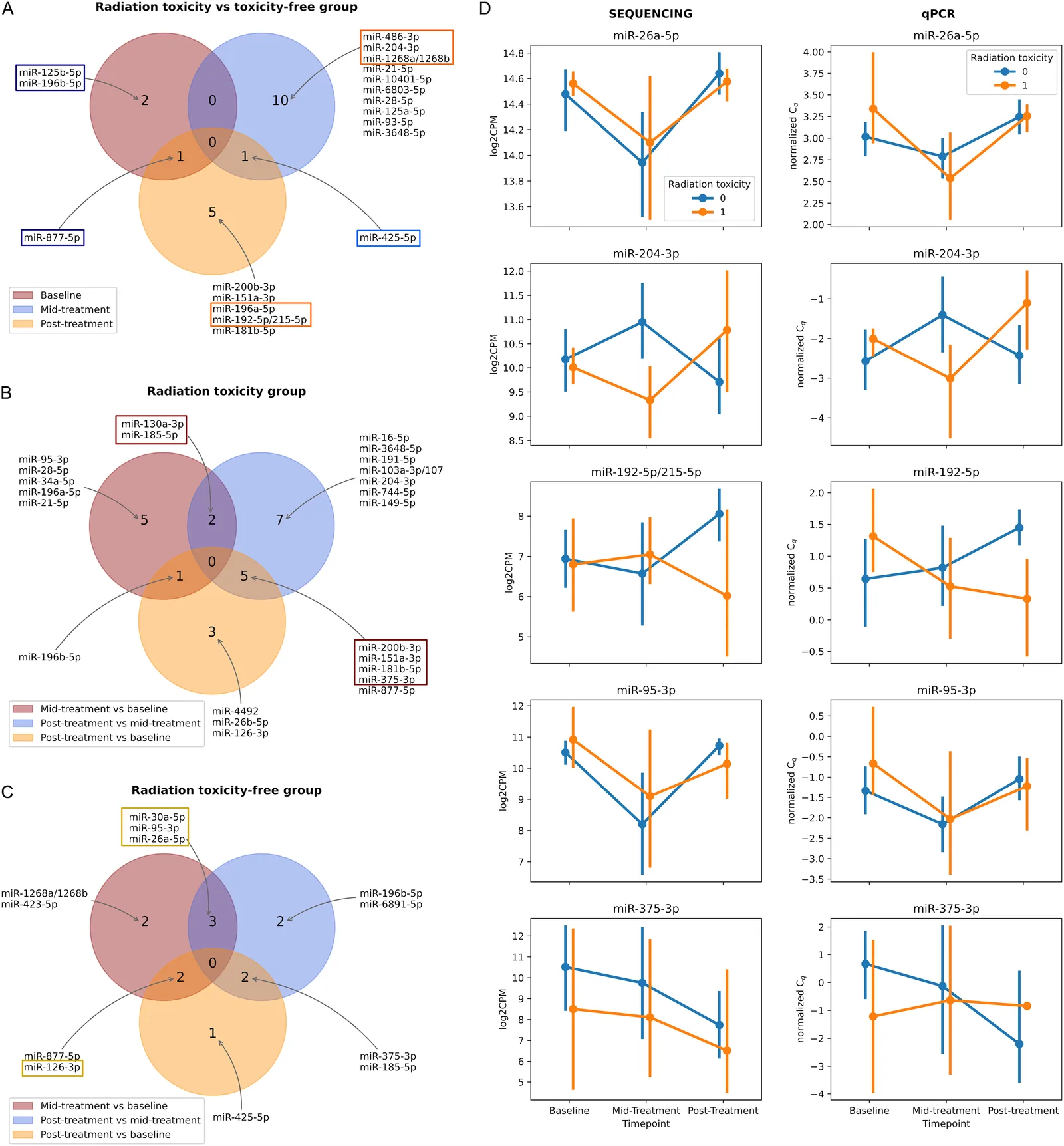
Venn diagrams showing miRNAs with significantly different expression levels between patients with and without urinary radiation toxicity at all timepoints. **(A)**, differences in miRNA expression between timepoints in patients with urinary radiation toxicity **(B)** and without urinary radiation toxicity **(C)**. Rectangular outlines show miRNAs selected for qPCR validation. miRNAs with similar expression patterns observed by sequencing (data represented as log-transformed counts per million) and qPCR (data presented as dCq calculated against Cq of reference miRNAs – higher values represent higher expression) **(D)**.

- Group 1: miR-877-5p, miR-125b-5p, miR-196b-5p (navy outline around names in **Figure 2A**).
- Group 2: miR-425-5p (blue outline **Figure 2A**).
- Group 3a: miR-130a-3p, miR-185-5p, miR-151a-3p, miR-181b-3p, miR-200b-3p, miR-375-3p (red outline in **Figure 2B**).
- Group 3b: miR-126-3p, miR-26a-5p, miR-95-3p, miR-30a-5p (yellow outline in **Figure 2C**).
- Group 4: miR-192-5p/215-5p, miR-196a-5p, miR-486-3p, miR-204-3p, miR-1268a/1268b (orange outline in **Figure 2A**).

We included also 3 reference miRNAs, identified by NormiRazor: let-7g, miR-574-5p, miR-98-5p as Group 0. It was the 12^th^ best among 13,244 possible combinations of 3 miRNAs (as 44 candidate miRNAs were identified) and the first one that did not contain any differentially expressed miRNA or members of their miRNA family (MIR30 family in this case, details in **Data Sheet 7**). However, qPCR data was finally normalized to let-7g only, since the remaining 2 candidates were expressed in too few samples to be considered as useful normalizers (data in **Data Sheet 8**).

Several findings from the miRNA-seq experiment were replicated by qPCR (full results of qPCR data analysis in **Table 2**). Expression of miR-95-3p dropped during radiation therapy (p<0.001 in qPCR and p<0.05 in sequencing in both groups), while later it started to normalize (**Figure 2D**). A similar effect was observed in the case of miR-26a-5p, however the decrease during radiotherapy was significant only in sequencing; in qPCR, miR-26a-5p showed a significant interaction between mid-treatment expression and toxicity (q=0.042). During MR-SBRT, expression of miR-204-3p showed a suggestive interaction with radiation toxicity (nominal p=0.010, q=0.087) where it decreased in patients experiencing urinary radiation toxicity, while it increased in the remaining ones. Findings for miR-192-5p, miR-375-3p, miR-486-3p, miR-1268s and miR-196a-5p (**Figure 2D**; **Supplementary Figure 4** in **Data Sheet 1**) overlapped partially between the two assays. miR-126-3p and miR-877-5p, that were identified as differentially expressed between patients with and without urinary radiation toxicity in the miRNA sequencing experiment, were detected in too few samples in qPCR (miR-877-p had missing expression in both replicates of 11 samples and single replicate of next 13, while miR-126-3p was not detected in any replicate of 19 samples and in only one replicate of further 3) to be evaluated.

**Table 2:** Variations in miRNA expression measured by qPCR.

miRNA	Mid-vs-Base	Post-vs-Base	Severity	RadTox	Mid: RadTox	Post: RadTox
hsa-miR-125b-5p	0.20 [-0.22, 0.61]	0.11 [-0.37, 0.58]	0.09 [-0.34, 0.52]	0.39 [-0.18, 0.96]	-0.33 [-0.98, 0.32]	-0.16 [-0.90, 0.58]
hsa-miR-1268a	1.31 [-0.10, 2.72]	-0.15 [-1.78, 1.48]	-0.34 [-1.73, 1.05]	-0.99 [-3.03, 1.05]	-0.66 [-3.04, 1.72]	1.19 [-1.39, 3.76]
hsa-miR-130a-3p	-0.34 [-0.83, 0.16]	0.27 [-0.29, 0.83]	0.36 [0.01, 0.71]	0.13 [-0.43, 0.69]	-0.59 [-1.37, 0.20]	0.46 [-0.42, 1.33]
hsa-miR-151a-3p	0.11 [-0.63, 0.85]	-0.06 [-0.99, 0.87]	0.55 [-0.06, 1.16]	0.34 [-0.55, 1.24]	-0.42 [-1.59, 0.75]	0.44 [-0.92, 1.80]
hsa-miR-181b-5p	0.44 [-0.33, 1.21]	0.30 [-0.53, 1.13]	0.74 [-0.03, 1.51]	-0.19 [-1.27, 0.88]	0.02 [-1.18, 1.22]	0.95 [-0.31, 2.22]
hsa-miR-185-5p	0.10 [-0.50, 0.70]	-0.65 [-1.34, 0.04]	-0.28 [-0.92, 0.36]	-0.36 [-1.26, 0.55]	0.32 [-0.70, 1.34]	1.08 [-0.02, 2.17]
hsa-miR-192-5p	0.17 [-0.64, 0.99]	0.75 [-0.18, 1.69]	0.48 [-0.22, 1.19]	0.67 [-0.34, 1.68]	-0.96 [-2.25, 0.33]	-1.58 [-3.05, -0.10]
hsa-miR-196a-5p	-0.44 [-1.03, 0.14]	0.01 [-0.58, 0.60]	0.89 [0.06, 1.72]	0.18 [-0.75, 1.12]	-0.56 [-1.40, 0.28]	0.38 [-0.54, 1.29]
hsa-miR-196b-5p	-0.35 [-0.98, 0.28]	-0.42 [-1.10, 0.26]	0.54 [-0.12, 1.19]	0.35 [-0.48, 1.17]	-0.15 [-1.11, 0.81]	0.73 [-0.46, 1.92]
hsa-miR-200b-3p	0.26 [-0.28, 0.80]	-0.10 [-0.72, 0.52]	0.24 [-0.44, 0.92]	0.21 [-0.62, 1.05]	0.11 [-0.73, 0.96]	0.30 [-0.66, 1.25]
hsa-miR-204-3p	1.17 [0.13, 2.21]	0.12 [-1.07, 1.31]	0.15 [-0.86, 1.16]	0.56 [-0.81, 1.94]	-2.17 [-3.81, -0.53]	0.86 [-0.98, 2.69]
hsa-miR-26a-5p	-0.23 [-0.46, 0.01]	0.15 [-0.13, 0.42]	0.34 [-0.13, 0.82]	0.32 [-0.20, 0.85]	**-0.57 [**-**0.94,** -**0.20]**	0.03 [-0.40, 0.45]
hsa-miR-30a-5p	-0.35 [-0.75, 0.06]	0.23 [-0.23, 0.70]	**0.84 [0.25, 1.43]**	0.45 [-0.24, 1.15]	-0.49 [-1.13, 0.14]	0.06 [-0.67, 0.79]
hsa-miR-375-3p	-0.18 [-2.09, 1.74]	-3.17 [-5.49, -0.86]	**-4.15 [-6.52, -1.78]**	-1.26 [-4.00, 1.47]	-1.50 [-5.07, 2.06]	0.61 [-3.96, 5.18]
hsa-miR-425-5p	0.48 [-0.07, 1.03]	0.09 [-0.54, 0.71]	0.25 [-0.13, 0.63]	0.05 [-0.57, 0.67]	-0.12 [-0.99, 0.75]	0.08 [-0.88, 1.04]
hsa-miR-486-3p	0.33 [-0.81, 1.46]	0.26 [-1.03, 1.56]	0.67 [-0.40, 1.74]	-0.76 [-2.36, 0.84]	-0.62 [-2.53, 1.29]	1.17 [-0.90, 3.23]
hsa-miR-95-3p	**-1.13 [-1.70, -0.56]**	0.03 [-0.53, 0.60]	0.64 [-0.64, 1.92]	0.67 [-0.69, 2.03]	-0.61 [-1.48, 0.25]	0.03 [-0.84, 0.91]
hsa-miR-98-5p	-0.49 [-1.11, 0.14]	0.18 [-0.43, 0.78]	0.48 [0.03, 0.94]	-0.06 [-0.75, 0.63]	0.87 [-0.04, 1.77]	0.16 [-0.78, 1.09]

Estimates from per-miRNA linear mixed models (ΔCt normalized to let-7g-5p) with fixed effects of timepoint, radiation toxicity, their interaction, and disease severity. Each cell shows the coefficient β (ΔCt scale) with 95% confidence interval; positive β indicates higher expression relative to the reference category (baseline, no toxicity, severity grade 0). p-values were adjusted across miRNAs within each effect (Benjamini-Hochberg); associations surviving FDR correction (q < 0.05) are bolded.

In qPCR, miR-30a-5p and miR-375-3p were found differentially expressed between patients with different degrees of disease severity (NCCN risk categories) at baseline (**Figure 3c**; **Supplementary Figure 5** in **Data Sheet 1**). In sequencing, disease severity was accountable for differences expression of 7 miRNAs (**Figures 3A, B**) with miR-375-p being the only one consequentially found by both techniques (**Figure 3C**).

**Figure 3:**
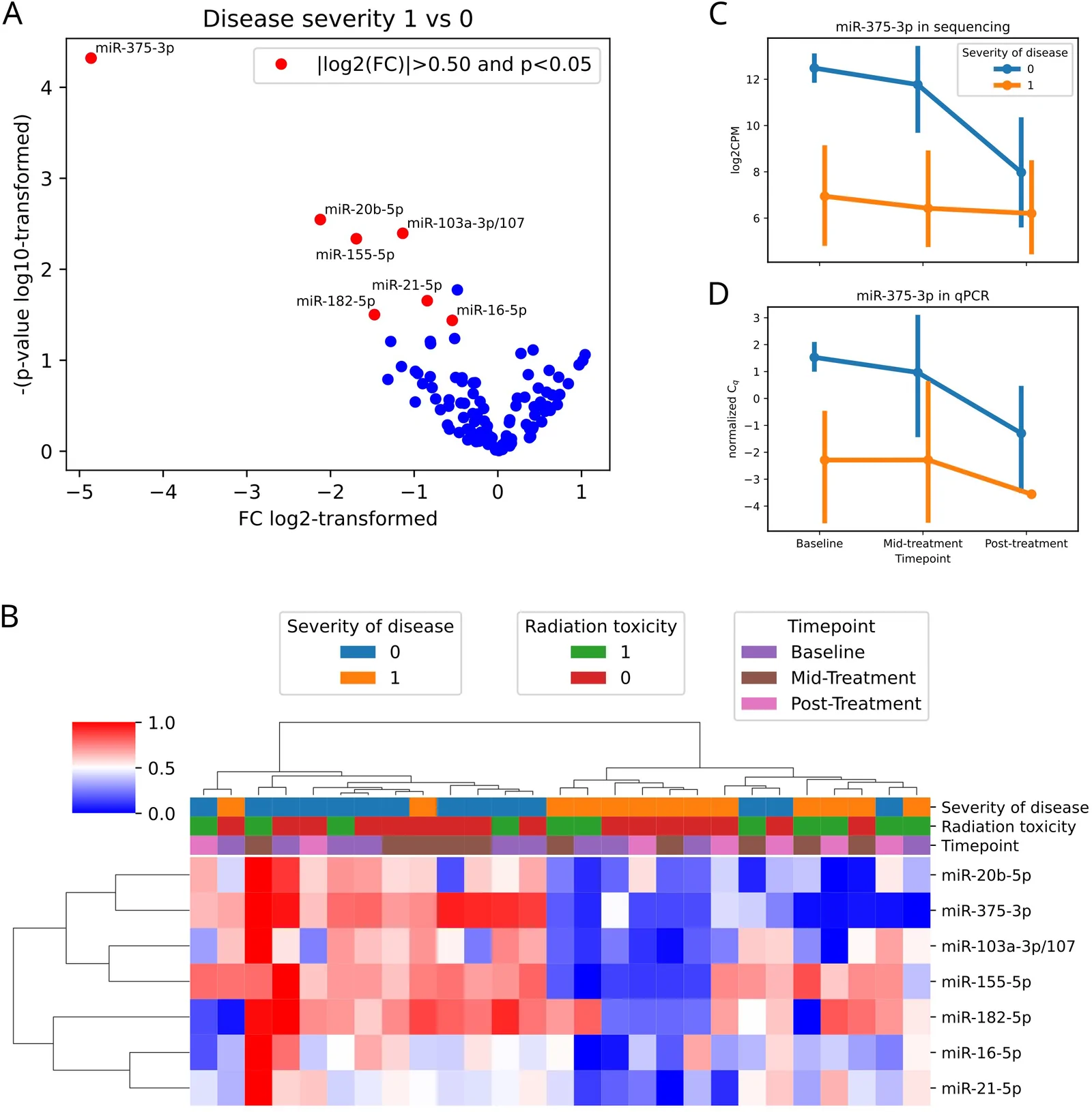
Effect of prostate cancer disease severity (0: CCN low to favorable intermediate risk, 1: NCCN unfavorable intermediate to high risk or recurrent/metastatic cancer) on miRNA expression with 7 miRNAs downregulated in severe disease marked in red **(A)** and shown in heatmap that demonstrates clustering by severity. Downregulation of miR-375-3p in severe observed in sequencing (data represented as log-transformed counts per million) **(C)** disease was confirmed in qPCR (data presented as dCq calculated against Cq of reference miRNAs – higher values represent higher expression) **(D)**.

The most significant pathways identified as enriched based on miRNAs significantly upregulated at mid-treatment timepoint in samples from patients with urinary radiation toxicity include the MAPK cascade, the NOD-like receptor signaling pathway and the IL1 signaling pathway. Meanwhile, no pathways were identified as enriched based on miRNAs significantly upregulated at mid-treatment timepoint in samples from patients without urinary radiation toxicity (**Table 3**).

**Table 3:** Top 10 pathways based on significant miRNAs upregulated in radiation effect = 1 vs 0 at mid-treatment​.

Subcategory​	Enrichment​	P-value​	P-adjusted​	miRNAs/precursors​
WP422 MAPK Cascade​	over-represented​	5.83E-05​	0.0214524​	hsa-miR-21-5p; hsa-miR-28-5p; hsa-miR-93-5p; hsa-miR-425-5p; hsa-miR-125a-5p​
hsa00310 Lysine degradation​	over-represented​	1.48E-04​	0.0273131​	hsa-miR-21-5p; hsa-miR-28-5p; hsa-miR-93-5p; hsa-miR-425-5p; hsa-miR-125a-5p​
hsa04621 NOD like receptor signaling pathway​	over-represented​	4.53E-04​	0.02814​	hsa-miR-21-5p; hsa-miR-28-5p; hsa-miR-93-5p; hsa-miR-425-5p; hsa-miR-125a-5p​
WP313 Signaling of Hepatocyte Growth Factor Receptor​	over-represented​	5.18E-04​	0.02814​	hsa-miR-21-5p; hsa-miR-28-5p; hsa-miR-93-5p; hsa-miR-425-5p; hsa-miR-125a-5p​
P04395 Vasopressin synthesis​	over-represented​	6.43E-04​	0.02814​	hsa-miR-21-5p; hsa-miR-93-5p; hsa-miR-125a-5p​
hsa04664 Fc epsilon RI signaling pathway​	over-represented​	6.71E-04​	0.02814​	hsa-miR-21-5p; hsa-miR-28-5p; hsa-miR-93-5p; hsa-miR-425-5p; hsa-miR-125a-5p​
hsa00230 Purine metabolism​	over-represented​	7.00E-04​	0.02814​	hsa-miR-21-5p; hsa-miR-28-5p; hsa-miR-93-5p; hsa-miR-425-5p; hsa-miR-125a-5p​
WP195 IL 1 signaling pathway​	over-represented​	7.60E-04​	0.02814​	hsa-miR-21-5p; hsa-miR-28-5p; hsa-miR-93-5p; hsa-miR-425-5p; hsa-miR-125a-5p​
hsa05216 Thyroid cancer​	over-represented​	9.30E-04​	0.02814​	hsa-miR-21-5p; hsa-miR-28-5p; hsa-miR-93-5p; hsa-miR-425-5p; hsa-miR-125a-5p​
hsa04912 GnRH signaling pathway​	over-represented​	0.0010052​	0.02814​	hsa-miR-21-5p; hsa-miR-28-5p; hsa-miR-93-5p; hsa-miR-425-5p; hsa-miR-125a-5p​

## Discussion

In this exploratory study, we identified urinary miRNAs whose expression levels were altered during radiotherapy for prostate cancer and differed between patients with or without radiation toxicity. These findings represent a promising proof of concept and feasibility study for future research developing non-invasive biomarkers for monitoring individual susceptibility to toxicity, potentially enabling more personalized radiotherapy approaches.

Importantly, our study is the first to assess miRNA expression in urine specifically in the context of prostate cancer radiotherapy toxicity. We confirmed our findings using two complementary methods—miRNA sequencing and quantitative PCR—which strengthens the reliability of our results. The use of urine as a non-invasive, easily accessible biofluid makes this approach particularly attractive for longitudinal monitoring in clinical settings, with potential applications in early identification of patients at risk for adverse effects and in adapting treatment strategies accordingly.

Several of the miRNAs identified as differentially expressed between timepoints or in patients with radiation toxicity in our study were previously linked to prostate cancer or radiation toxicity. For example, miR-95-3p, which was differentially expressed in urine mid-treatment vs baseline, was suggested to promote the development of prostate cancer via regulating DKK3 and activating Wnt/β-catenin pathway (13). In cervical cancer, its high expression was associated with poor prognosis and mechanistically linked to promotion of malignant behavior via repression of VCAM1 expression (14). Knockdown of miR-95-3p in non-small lung cancer increased radiosensitivity, while its elevated levels were observed in recurrent disease (15).

MiR-30a-5p, which in our analysis showed higher urinary expression in patients with more advanced disease, was found to be increased in expression in peripheral blood after total body irradiation. Radiation was also reported to reduce miR-30a-5p expression (16) inLNCaP cells while its elevation sensitized LNCaP cells to radiation via TP53INP1 suppression. A nominal association with disease severity was also noted for miR-130a-3p. Previously, serum miR-130a-3p levels have been shown to be increased in peripheral blood after irradiation in mice (17). Also, miR-130a-3p levels are downregulated in radiotherapy-sensitive patients with NSCLC (18).

In our study, both in the microRNA sequencing and qPCR analysis, miR-375-3p showed higher expression in patients with less advanced cancers. On the other hand, prostate cancer patients had higher levels this miRNA than men with benign hyperplasia, but with limited diagnostic performance (19). Previously, higher expression of miR-375-3p in peripheral blood was found after total body irradiation (5) and in a meta-analysis of miRNAs biomarkers of radiation exposure (4); further, miR-375 in the plasma were associated with significantly poorer overall survival in patients with castrate-resistant metastatic PCa (20).

We found also that miR-192-5p showed a suggestive association with radiation toxicity post-treatment, which was however not significant after multiple comparisons correction. While no reports yet have linked miR-192-5p to radiation response, it was described as overexpressed and suggested to promote prostate cancer cell proliferation (21).

Pathway enrichment analysis confirms the involvement of identified miRNAs in processes relevant for radiation-induced side effects. One of the most enriched based on miRNAs significantly upregulated at mid-treatment timepoint in samples from patients with radiation toxicity was NOD-like receptor signaling pathway that mediates the release of inflammatory factors and, *via* nucleotide-binding domain and leucine-rich-repeat-containing family pyrin 3 (NLRP3) inflammasome, is crucially involved in radiation-induced tissue and organ damage (22).

Several limitations of the present study should be mentioned. First, the limited number of participants restricts the statistical power of the study, increasing the risk of both type I and type II errors. The analysis involved multiple comparisons across a panel of candidate miRNAs, raising the possibility of false discoveries that will require verification in larger studies planned to strictly control multiple comparisons. Although most results were replicated by both sequencing and qPCR, the risk of type I error remains, especially given the exploratory nature of the study. Importantly, several miRNAs identified as differentially expressed in sequencing could not be assessed by qPCR due to low detection rates in urine, highlighting platform concordance limitations and the need for improved assay sensitivity in future studies.

Further, our study exclusively assessed early radiotherapy-induced toxicity (CTCAE grade ≥2 within 3 months), and did not address the occurrence or molecular correlates of late toxicity, which can be clinically significant. Notably, all four patients who experienced urinary toxicity received tamsulosin in response to their symptoms, whereas none of the toxicity-free patients did. As medication use and toxicity status were therefore confounded, we cannot exclude the possibility that some of the observed between-group differences in miRNA expression reflect tamsulosin exposure rather than radiation toxicity itself. Also, given a relatively heterogenous cohort of patients, the findings may not be generalizable to broader patient populations. Due to sample size and missing data in clinical records, some potentially important clinical confounders (e.g. concomitant diseases) could not be included in present analysis. As recent report by Satomura et al. (23) shows that miRNA yields in urine depend on time of collection and urine’s biophysical characteristic, stricter standardization of collection protocol would be advisable.

Future studies will be needed to clarify if observed association between miRNA expression and radiation toxicity are causal, since some differentially expressed miRNAs were already implicated in other biological processes. For instance, miR-877–5 was repeatedly reported as tumor suppressor in prostate cancer (24, 25) and hepatocellular carcinoma (26), while in our study its levels change oppositely in patients who did and did not experience radiation toxicity. Similarly, miR-26a-5p is considered a tumor suppressor targeting *SERBP1* in prostate cancer (27) and inhibiting WNT5A/β-catenin signaling in the urothelial carcinoma cell line (28). High expression of miR-204, that in our study increases during RT in toxicity-free group, sensitized prostate cancers cells to doxorubicin (29), and inhibited proliferation of bladder cancer cells (30).

Given these limitations, future research should prioritize validation of candidate urinary miRNA biomarkers in larger and more homogeneous patient cohorts. Studies should aim to evaluate both early and late radiotherapy-induced toxicities, and systematically account for confounding variables such as disease stage and ADT exposure. Ultimately, prospective, multi-institutional studies are needed to clarify the clinical utility of urinary miRNAs for predicting and monitoring treatment-related toxicity, and to support the development of personalized radiotherapy strategies.

We conclude that miRNA profiling in urine samples is feasible and may provide a novel diagnostic and prognostic modality in prostate cancer if validated in larger cohorts. In this exploratory pilot study, we identified urine microRNAs that may be associated with the development of radiation-associated urinary toxicities and severity of prostate cancer, which warrants further exploration.

## Data Availability

Data generated for this study have been deposited in Gene Expression Omnibus under accession number GSE300942. All other data is provided within the manuscript and supplementary information files. For questions on the study data please contact Urszula Smyczyńska at urszula.smyczynska@umed.lodz.pl.
