# Supplementary material for: A prospective pilot study of urine microRNA as a biomarker of urinary radiation toxicity following MRI-guided prostate stereotactic body radiotherapy
Source: Front Oncol. 2026 Oct 7;16:1951947. doi: 10.3389/fonc.2026.1951947 (PMC13683214; doi:10.3389/fonc.2026.1951947)
Supplement: Supplementary file 1 [file DataSheet9.pdf]

Supplementary methods

Bioinformatic miRNA sequencing analysis

Raw data was de-multiplexed, followed by FASTQ files generation for each sample with the bcl2fastq2 software (Illumina inc.). Reads from FASTQ files were mapped to miRBase (version 22) using miRge3.0 software [1] (Patil & Halushka, 2021) with UMI deduplication. Due to low total count, mapping was repeated using nf-core/smrnaseq (version 2.2.1) pipeline [2] without UMI deduplication to confirm counts were not overly reduced by deduplication.

Data filtration was applied so that only miRNAs with at least 100 counts per million (CPM) In at least half of the samples were left for further analysis. Then, normalization was performed with voom [3], comprising mean-variance trend removal, transformation to counts per million, logarithmic (log2) transform. Finally, we performed differential expression (DE) analysis using limma [4]. We included effects of disease severity, urinary radiation toxicity, time point, and interaction between the latter two (model formula: miRNA ~ Severity + Timepoint*Radiation Effect). Interaction term was included as we assumed that urinary radiation toxicity effects can develop over time. Effect of androgen deprivation therapy was included in initial model, too, but was removed from final version as no miRNA passed significance threshold for this effect (Suppl. Fig. 7). Repeated measurements in the same patients have been taken into account using duplicateCorrelation function from limma package. Differences with nominal p-value<0.05 and |log2(fold-change)| > 0.5 were considered significant. Principal component analysis (PCA) was used for data exploration and visualization.

Supplement: miRNA selection criteria for qPCR.

miRNAs for qPCR experiment were prioritized by the following criteria:

- Group 0 - reference miRNAs identified by NormiRazor: let-7g, miR-574-5p, miR-98-5p
- Group 1 - miRNAs that at baseline differed between patients with and without radiation toxicity: miR-877-5p, miR-125b-5p, miR-196b-5p (navy outline around names in Fig. 2A).
- Group 2 – miRNAs that differed between patients with and without radiation toxicity mid-treatment and post-treatment: miR-425-5p (blue outline Fig. 2A)
- Group 3a – miRNAs that differed in at least 2 between timepoints comparisons in patients with radiation toxicity: miR-130a-3p, miR-185-5p, miR-151a-3p, miR-181b-3p, miR-200b-3p, miR-375-3p (red outline in Fig. 2B)
- Group 3b – miRNAs that differed in at least 2 between timepoints comparisons in patients without radiation toxicity: miR-126-3p, miR-26a-5p, miR-95-3p, miR-30a-5p (yellow outline in Fig. 2C)
- Group 4 – selected miRNAs that differed between patients with and without radiation toxicity mid- or post-treatment: miR-192-5p/215-5p, miR-196a-5p, miR-486-3p, miR-204-3p, miR-1268a/1268b (orange outline in Fig. 2A)

Supplementary Tables

Supplementary Table 1. Individual data of participants included in the study.

Patient ID	Age	Gleason grade group	NCCN risk category	Received androgen deprivation therapy	Prostate volume (cc)	Bladder V36.25 (cc)	Bladder V32,62 (cc)	Bladder V38,06 (cc)	Urethra V38.78 (cc)	Urinary medications	Radiation toxicity	Interval from end of RT to follow-up sample (days)	Baseline AUA/IPSS
1	56	2	Favorable Intermediate Risk	No	29.6	0.4	3.01	0.1	0	Tamsulosin	Yes	104	12
2	69	2	Favorable Intermediate Risk	No	66	3.5	15.3	0.06	0	None	No	97	10
3	75	2	Favorable Intermediate Risk	Yes	67.4	1.5	8.75	0	0	Tamsulosin	Yes	83	4
4	73	2	Favorable Intermediate Risk	No	89.1	4.79	14.32	0	0	None	No	115	11
5	59	1	Low Risk	No	82.5	2.9	9.96	0	0	None	No	90	5
6	79	3	Unfavorable Intermediate Risk	Yes	29.4	2.2	9.75	0	0	None	No	87 (not sequenced)	1
7	75	3	High Risk	Yes	19.9	0	0.18	0	0	None	No	77	10
8	65	4	Metastatic	Yes	36	1.7	9.4	0	0	Tamsulosin	Yes	107	N/A
9	89	3	High Risk	Yes	21	3.4	11.18	0.1	0	Tamsulosin	Yes	N/A	9
10	64	4	High Risk	No	20.4	0	0.5	0	0	None	No	N/A	6

Supplementary Table 2: Spearman correlations between baseline miRNA expression and AUA/IPSS scores

miRNA	r	p
miR-425-5p	-0.13	0.7313
miR-26a-5p	-0.18	0.6354
miR-95-3p	-0.01	0.9830
miR-30a-5p	0.30	0.4308
miR-185-5p	-0.03	0.9319
miR-375-3p	0.18	0.6511
miR-126-3p	-0.31	0.4175
miR-196b-5p	0.18	0.6511
miR-21-5p	0.28	0.4581
miR-130a-3p	-0.38	0.3063
miR-151a-3p	0.18	0.6511
miR-181b-5p	0.04	0.9149
miR-200b-3p	0.35	0.3537
miR-196a-5p	-0.03	0.9319
miR-192-5p/215-5p	-0.06	0.8810
miR-204-3p	-0.44	0.2318
miR-125b-5p	-0.18	0.6511
miR-486-3p	-0.41	0.2730
miR-1268a/1268b	-0.47	0.2032

Supplementary Figures

Supplementary Figure 1: Differences in miRNA expression observed between patients with and without radiation toxicity at baseline (A), mid-treatment (B) and post-treatment (C). miRNAs from groups 1 and 2 (D).

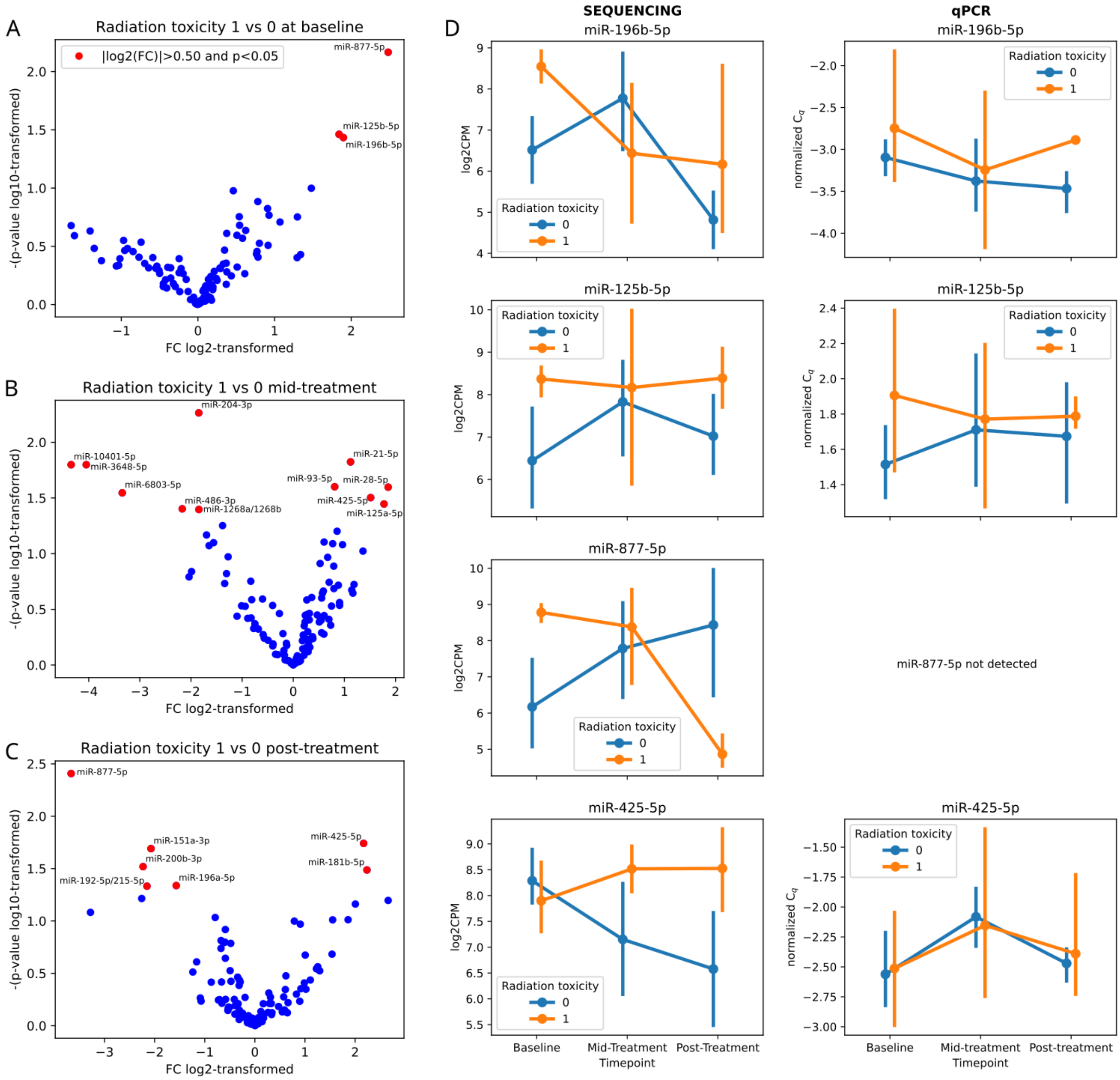

Supplementary Figure 2. Differences in miRNA expression observed between timepoint in patients with radiation toxicity (A, B, C). miRNAs from group 3a.

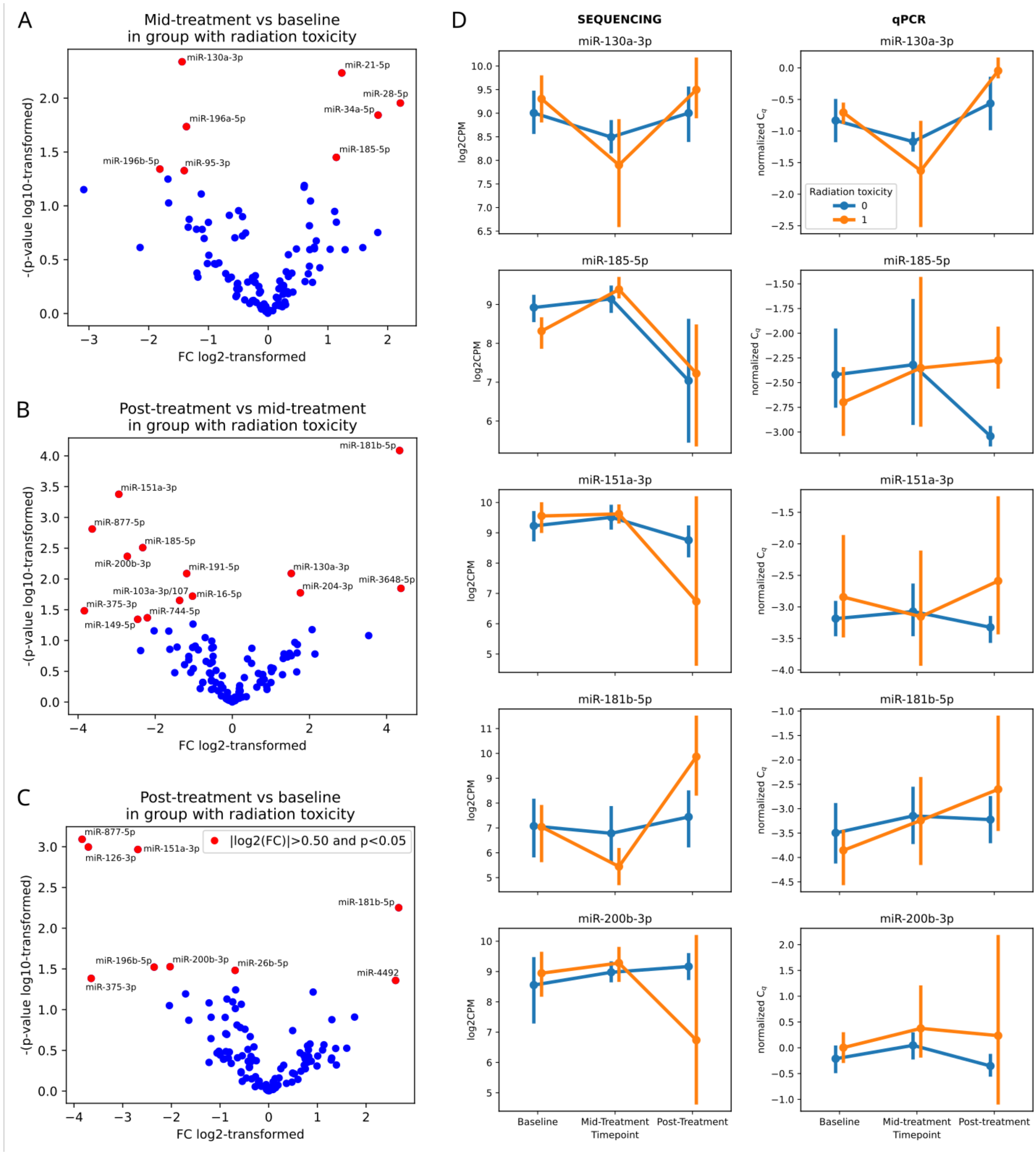

Supplementary Figure 3. Differences in miRNA expression observed between timepoint in patients without radiation toxicity. miRNAs from group 3b.

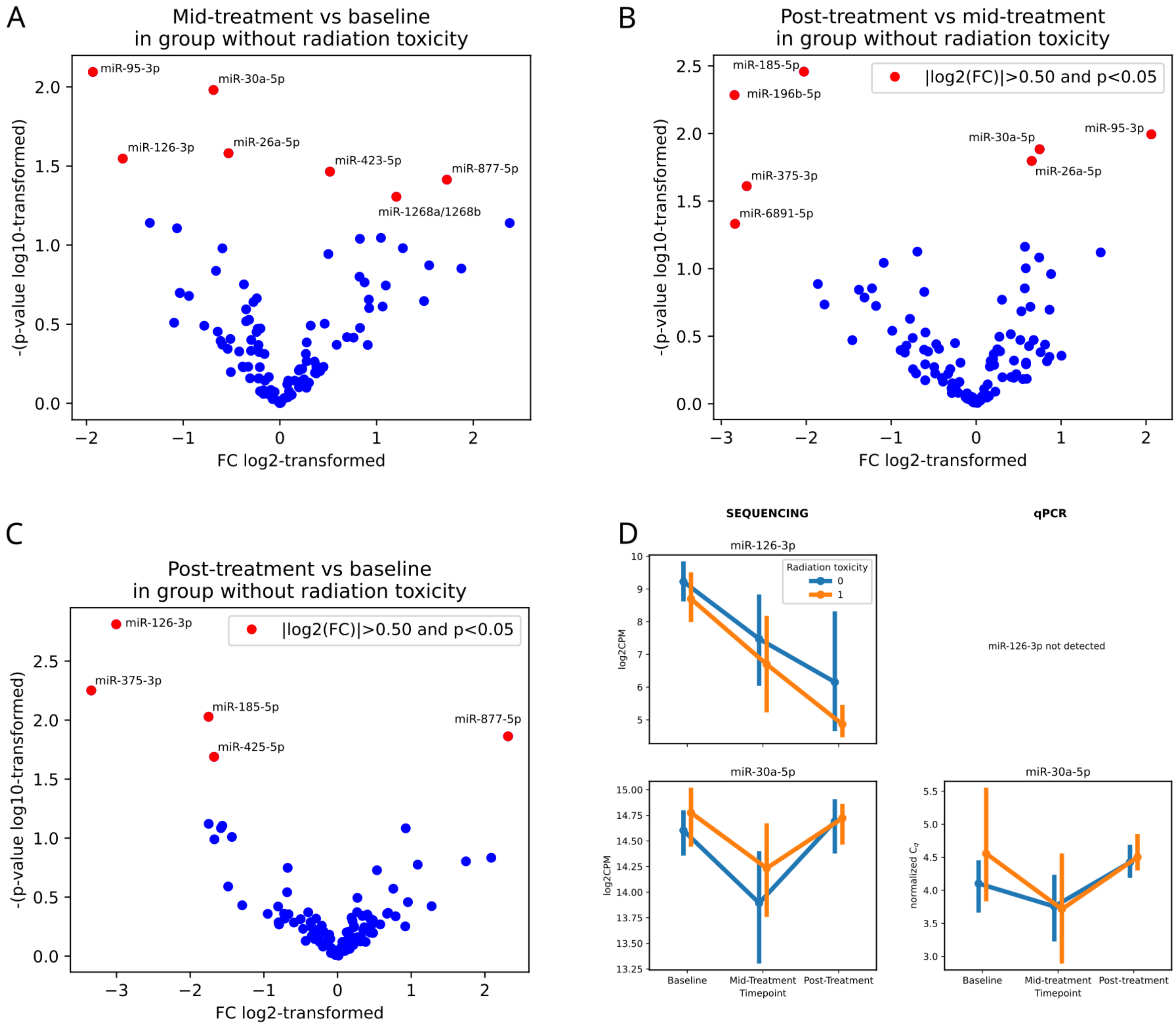

Supplementary Figure 4. Remaining miRNAs from group 4.

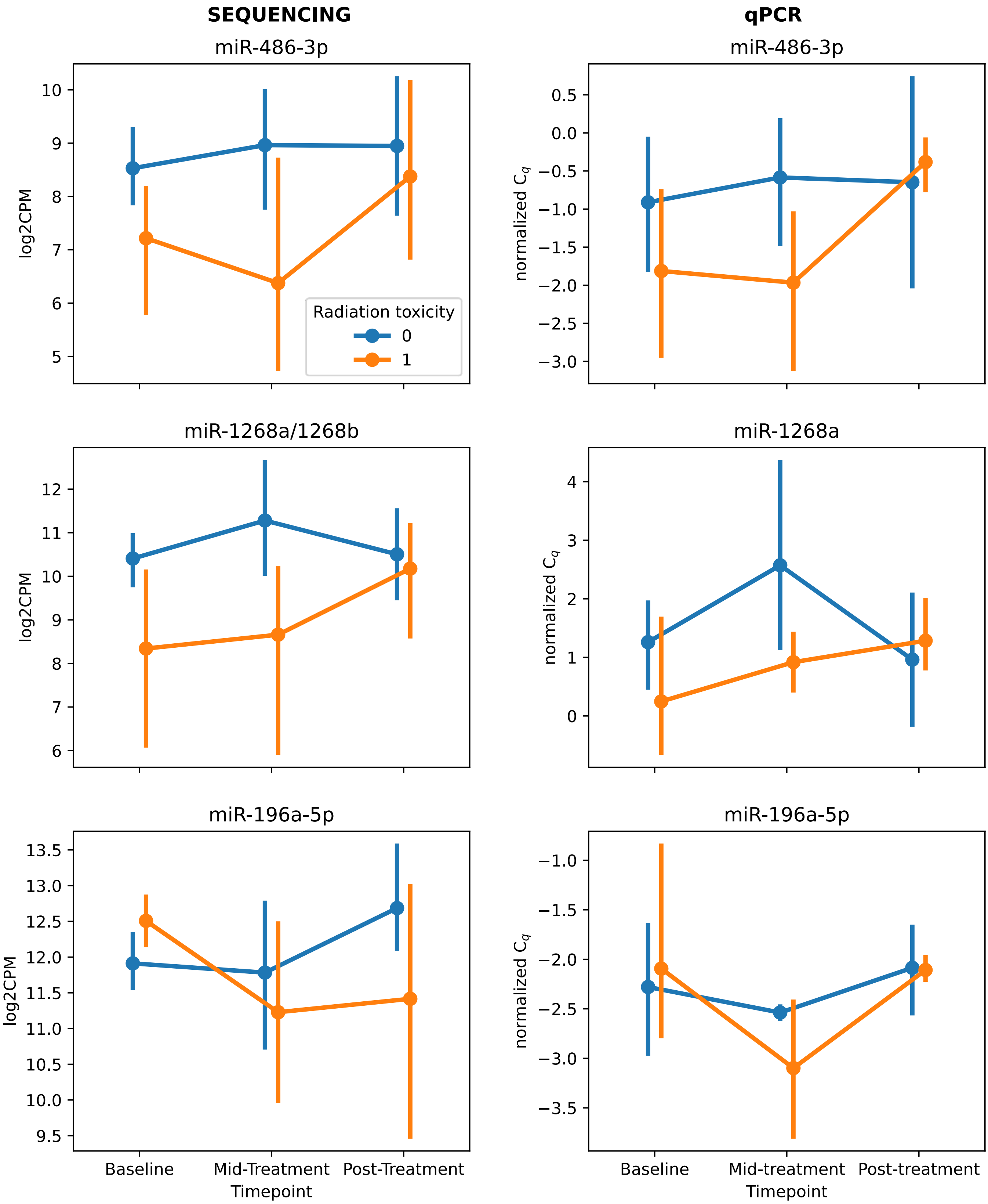

Supplementary Figure 5. miRNAs with differential expressed between patient with more and less severe disease only in qPCR analysis.

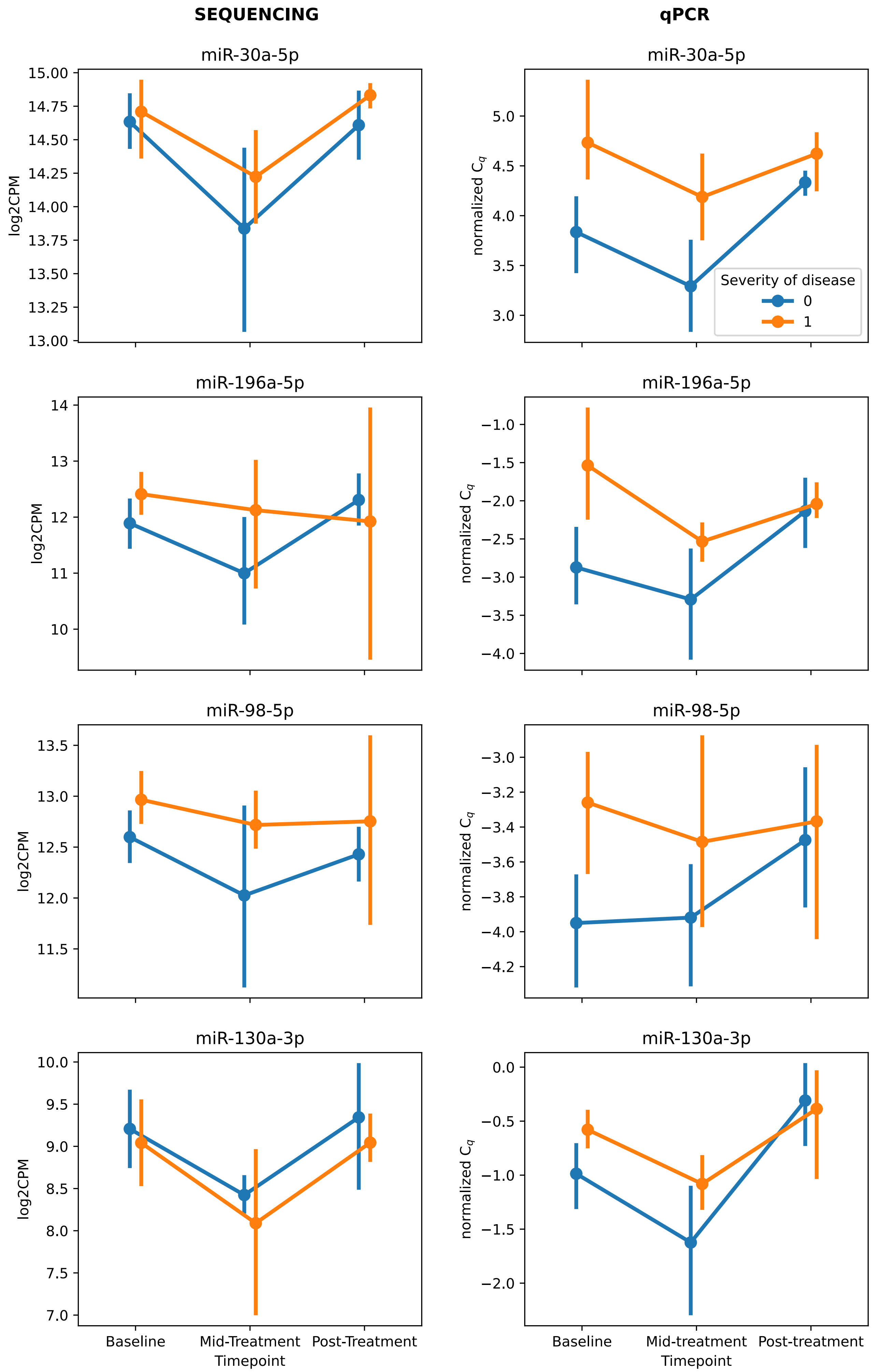

Supplementary Figure 6: Stability of let-7g-5p with respect to timepoint, radiation toxicity and severity of disease.

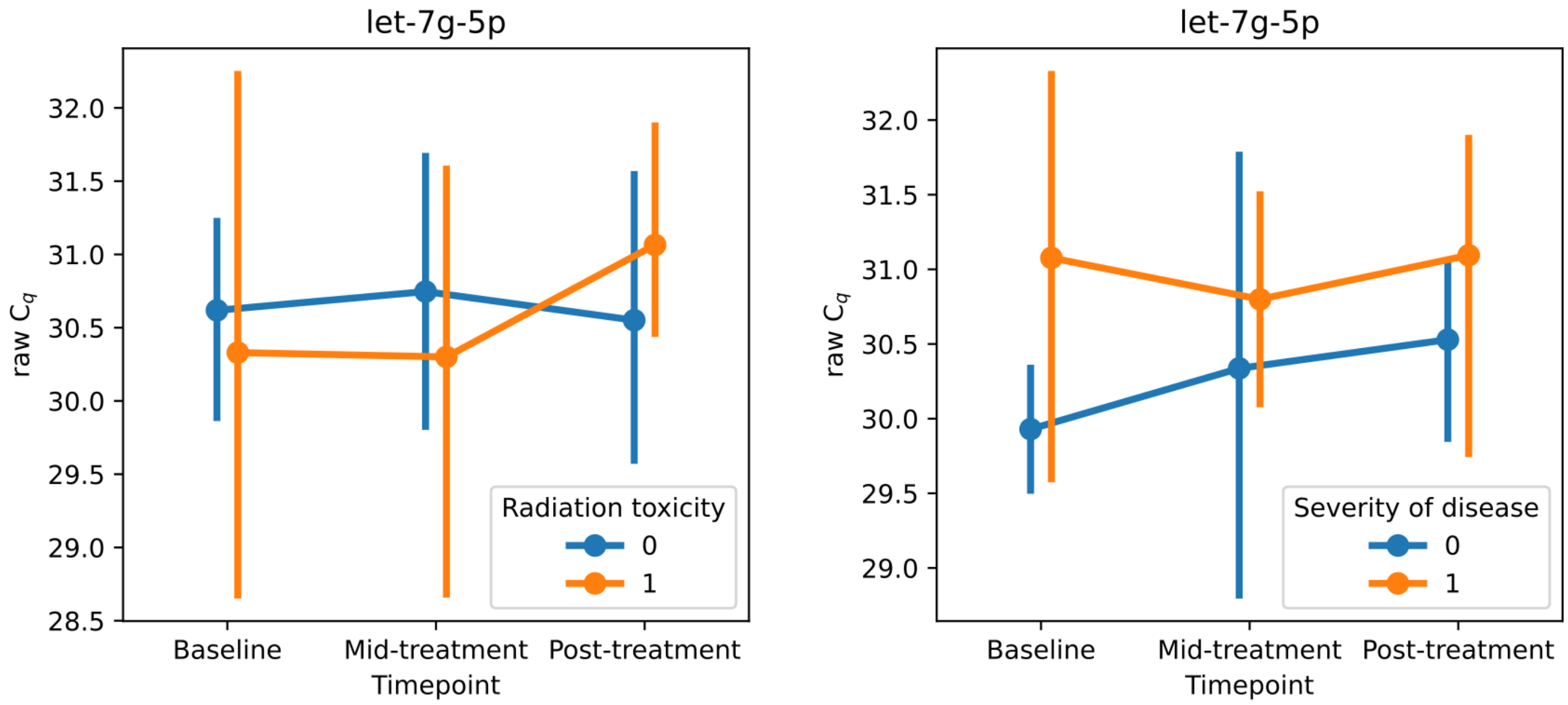

Supplementary Figure 7: Effect of androgen deprivation therapy (ADT) on miRNA expression

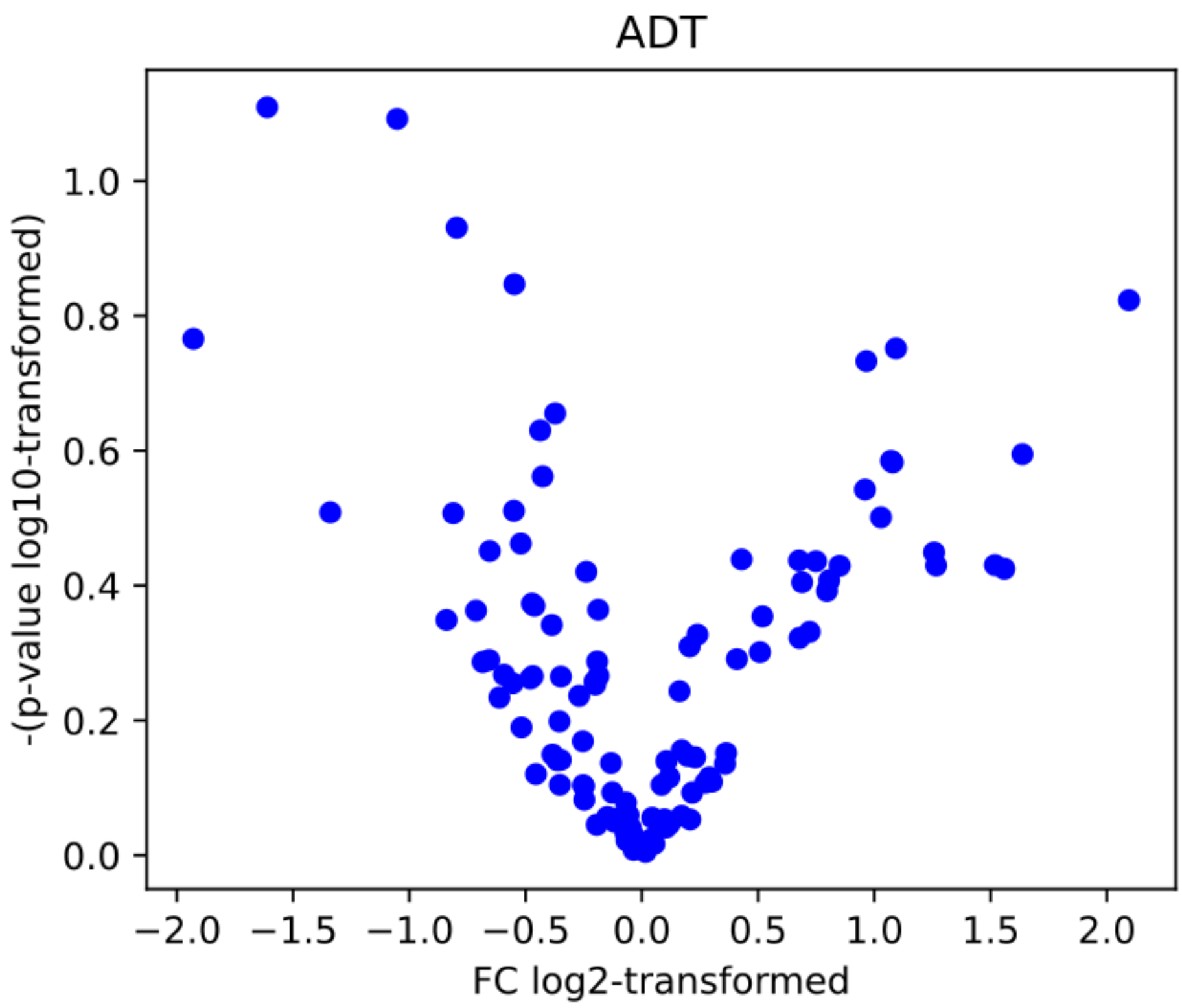

Supplementary references

[1] Patil AH, Halushka MK. miRge3.0: a comprehensive microRNA and tRF sequencing analysis pipeline. NAR Genom Bioinform 2021;3:lqab068. https://doi.org/10.1093/nargab/lqab068.

[2] nf-core/smrnaseq 2024.

[3] Law CW, Chen Y, Shi W, Smyth GK. voom: Precision weights unlock linear model analysis tools for RNA-seq read counts. Genome Biol 2014;15:R29. https://doi.org/10.1186/gb-2014-15-2-r29.

[4] Ritchie ME, Phipson B, Wu D, Hu Y, Law CW, Shi W, et al. limma powers differential expression analyses for RNA-sequencing and microarray studies. Nucleic Acids Res 2015;43:e47. https://doi.org/10.1093/nar/gkv007.
